# DPP-4 inhibitors in drug-resistant epilepsy: a hypothesized mechanism via the gut microbiota-short-chain fatty acids-glucagon-like peptide-1 axis

**DOI:** 10.3389/fimmu.2026.1860661

**Published:** 2026-06-23

**Authors:** Xili Fu, Yinyin Xie, Yi Xie, Aoya Han, Xinru Zhou, Shijie Zhang, Changchang Shen, Yuxuan Zhang, Lina Wang, Nanchang Xie

**Affiliations:** Department of Neurology, The First Affiliated Hospital of Zhengzhou University, Zhengzhou, China

**Keywords:** DPP-4 inhibitors, drug-resistant epilepsy, GLP-1, gut microbiota, gut–brain axis, short-chain fatty acids

## Abstract

**Background:**

Drug-resistant epilepsy (DRE) affects approximately one-third of patients with epilepsy and remains a major therapeutic challenge.Recent studies have demonstrated significant gut microbiota dysbiosis in patients with DRE, and certain interventions targeting the gut microbiota demonstrate therapeutic efficacy. However, pharmacological interventions that precisely modulate the gut microbiota in DRE have not yet been fully explored.

**Objective:**

This review aims to propose a systematic hypothesis that Dipeptidyl peptidase-4 inhibitors (DPP-4is) may alleviate peripheral and central pathological damage by regulating the “gut microbiota-short-chain fatty acids (SCFAs) -glucagon-like peptide-1 (GLP-1) axis”, thereby reducing susceptibility to DRE.

**Evidence:**

Existing studies indicate that: (1)DPP-4is possess neuroprotective effects in experimental epilepsy models, partly by enhancing endogenous GLP-1 signaling. (2)DPP-4is have been reported to modulate gut microbiota composition and increase the abundance of SCFA-producing bacteria in metabolic diseases. (3)SCFAs can promote GLP-1 secretion by activating free fatty acid receptors (FFAR2/3) and improve intestinal barrier function and inflammatory status in metabolic and neurodegeneration disease. However, it remains unclear whether this pathway mediates the effects of DPP-4is in epilepsy. (4)Enhanced peripheral GLP-1 signaling can further influence central nervous system homeostasis, including enhancing inhibitory synaptic transmission, attenuating neuroinflammation, oxidative stress, and inhibiting neuronal apoptosis, thereby reducing susceptibility to seizures.

**Conclusion:**

By integrating cross-contextual evidence, we propose that DPP-4is may exert protective effects on DRE through gut microbiota-SCFAs-GLP-1 axis.

## 1 Introduction

Drug-resistant epilepsy (DRE) is defined as the failure to achieve seizure control after adequate trials of two anti-seizure medications (ASMs). Approximately one-third of patients eventually develop DRE, and existing ASMs demonstrate limited efficacy against common epilepsy comorbidities, such as cognitive impairment. Therefore, there is an urgent need to identify novel intervention targets for DRE treatment. In recent years, the gut microbiota has been found to influence the onset and progression of various neurological diseases via the gut-brain axis. Evidence indicates that the gut microbiota of patients with DRE differs significantly from that of drug-sensitive patients and healthy controls (1).Meanwhile, microbiota-targeted interventions, such as the ketogenic diet, probiotics, and fecal microbiota transplantation, have demonstrated potential benefits in some DRE patients (2). However, existing gut microbiota interventions often lack precise targets, and treatment outcomes may vary significantly among individuals. Therefore, identifying pharmacological approaches that can more specifically modulate the gut microbiota may present new opportunities for DRE treatment.

Dipeptidyl peptidase-4 inhibitors (DPP-4is) are a class of drugs widely used in the treatment of type 2 diabetes. Their classical action is to inhibit DPP-4-mediated rapid inactivation of glucagon-like peptide-1 (GLP-1), thereby enhancing endogenous GLP-1 signaling (3). GLP-1 is primarily secreted by intestinal L cells, and its receptor belongs to the class B G protein-coupled receptors, which are widely expressed on the surfaces of brain neurons and glial cells (4). Beyond their glucose-lowering effects, a growing body of research indicates that DPP-4is can participate in the regulation of central inflammation, oxidative stress, apoptosis, and synaptic homeostasis by enhancing endogenous GLP-1 activity (5), and exert neuroprotective effects in neurodegenerative diseases and experimental epilepsy models (6–8). Notably, DPP-4is (sitagliptin) have been reported to reduce seizure frequency in a dose-dependent manner in a mouse model of DRE (9), and this effect can be attenuated by GLP-1 receptor antagonists (6), suggesting that GLP-1 signaling may be a key mediator of the central neuroprotective effects of DPP-4is.

However, current explanations for the antiepileptic effects of DPP-4is primarily focus on central GLP-1 signaling. Given that most DPP-4is have limited ability to cross the blood-brain barrier (BBB) (10), it is necessary to investigate the peripheral mechanisms by which DPP-4is regulate GLP-1 secretion. Given the significant role of gut dysbiosis in DRE, recent research on gastrointestinal diseases has shown that DPP-4is can improve gut microbiota balance, especially increasing the short-chain fatty acid (SCFA)-producing bacteria (11). Further studies have shown that SCFA-producing bacteria may serve as an imprint associated with epilepsy (12). What’s more, it can promote GLP-1 secretion by binding to G protein-coupled receptors in the gut (13).

Based on this, we propose an integrative framework that DPP-4is may serve as a potentially novel treatment for DRE through the hypothetical “gut microbiota-SCFAs-GLP-1” pathway. Direct evidence regarding this axis remains limited. Therefore, this review aims to summarize the existing evidence, identify gaps in the mechanistic understanding and provide a cautious framework for future validation.

## 2 Gut Microbiota and DRE

### 2.1 Gut microbiota characteristics in DRE patients

Previous studies have shown that both animal models of epilepsy and human patients exhibit significant alterations in gut microbiota compared with healthy controls (14), suggesting an important role of the gut microbiota in epileptogenesis and potential therapeutic value. Therefore, we summarize studies from the past eight years investigating gut microbiota alterations in DRE patients(**Table 1**). As shown in the table, the heterogeneity in α- and β-diversity of the gut microbiota in DRE across different studies may be influenced by confounding factors such as the participants’ age, diet, medication use and sample size. What’s more, the DRE patients generally exhibit upregulation of phylum Firmicutes (including the class Negativicutes and the genus *Hungatella*) and the phylum Proteobacteria; simultaneously, there is downregulation of beneficial bacteria such as the genus *Bacteroides, Bifidobacterium, Ruminococcus_g2*, *Eubacterium*, and the species *Bacteroides finegoldii*. Interestingly, these observed bacteria which are decreased in abundance in DRE are all major producers of SCFAs (such as butyrate, propionate, and acetate) (15), suggesting that decreased SCFA levels in the body may be associated with DRE. Studies have shown that SCFAs are involved in regulating intestinal inflammation, barrier function, and endocrine functions (such as GLP-1 secretion), and can further influence central nervous system homeostasis, making them potential therapeutic targets in DRE.

**Table 1:** Overview of previous studies (2017-2025) about gut microbiota in DRE.

Study design	Sample size	Age mean(range)	ASM status	Effects direction(high abundance↑/low abundance ↓)	p values	References
cross-sectional case- control study	DR(20) vs DS(21) vs HC(27)	7.2(2.6-11.8)	**DR:**drug-resistant, -DRE-DR:drug responsive DRE(a reduction in seizure frequency of at least 75% over one year) -DRE-DNR:drug non-responsive DRE **DS:**drug-sensitive,**HC:**healthy control	**α-diversity:**no significant difference among three **β-diversity:**significant difference (DR/DS vs HC)^1^ **Genus:** *Hungatella*↑(DR vs HC)^2^; *Eubacterium*↓(DR vs DS)^3^	p^1^ = 0.001 0.01≤p^2^<0.05 0.01≤p^3^<0.05	(1)
retrospective case-control study	DR(14) vs HC(30)	1.95(0.8–3.3)		**lower α-diversity** **β-diversity:**clearly distinguished **Phylum:** Firmicutes, Proteobacteria↑; Bacteroidetes, Actinobacteria↓ **Genus:** *Cronobacter*↑; *Bacteroides*, *Prevotella*, *Bifidobacterium*↓	p:not clear	(17)
prospective case-control study	DR(42) vs DS(49) vs HC(65)	28.4 (5–50)		**higher α-diversity**(DR vs DS/HC)^1^ **β-diversity:**different(DR vs DS) **Phylum**(DR vs DS):Firmicutes, Verrucomicrobia↑; Bacteroidetes↓ **Genus**(DR vs DS):rare genera*↑; *Bacteroides*, *Barnesiell*↓in DR, *Bifidobacteria*, *Lactobacillus*↓in DR(with higher frequency≥4 seizures per year)	p^1^ = 0.03 other p:not clear	(18)
prospective case-control study	DR(8) vs HC(32)	3.17 (1.16–6.92)	**DR:**drug-resistant, -DRE-DR:drug responsive DRE(a reduction in seizure frequency of at least 75% over one year) -DRE-DNR:drug non-responsive DRE **DS:**drug-sensitive,**HC:**healthy control	**lower α-diversity**^1^ **β-diversity:**significant difference^2^ **Phylum:** Actinobacteria↑; Bacteroidetes↓ **Family:** Actinomycetaceae, Bifidobacteriaceae, Coriobacteriaceae, Corynebacteriaceae, Enterococcaceae↑	p^1^<0.01 p^2^ = 0.001 other p:<0.01	(19)
prospective case-control study	DR(20) vs DS(20)	41(27.4-54.6)		**overall diversity:** no significant difference **Class:** Negativicutes(which belong to Firmicutes)↑ **Genus:** *Ruminococcus_g2*↓ **Species:** *Bacteroides finegoldii*↓	p:not clear	(20)
meta- analysis	DR(183) vs HC(283)	not clear		**overall diversity:** not reported **Phylum:** Proteobacteria^1^, Verrucomicrobia^2^↑; Bacteroidetes^3^↓ **Family:** Ruminococcaceae^4^(which belong to Firmicutes)↓	p^1^ = 0.04 p^2^ = 0.01 p^3^ = 0.03 p^4^<0.00001	(21)
cross-sectional case-control study	DR(29,DRE-DR=13,DRE-DNR=16) vs DS(31) vs HC(36)	(3-18)		**α-diversity:**higher species richness(DR vs HC^1^,DR vs DS^2^), no difference in evenness among three **β-diversity:**significant difference(DR/DS vs HC)^3^ **Class**(DR vs DS):Saccharimonadia^4^↓ **Order**(DR vs DS):Saccharimonadales^5^↓ **Family**(DRE-DNR vs DRE-DR):Rhodobacteraceae^6^,Vibrionaceae^7^↑; Leuconostocaceae^8^↓ **Genus:-**(DRE-DNR vs DRE-DR) *Enterobacter*^9^,*Grimontia*^10^↑; -(DR vs DS) *Peptoclostridium*^11^, *Saccharimonadales*^12^↓	p^1^<0.01 p^2^<0.05 q^3^ = 0.001 p^4^<0.0001 p^5^<0.0001 p^6^ = 0.0002 p^7^ = 0.0025 p^8^ = 0.0008 p^9^<0.0001 p^10^<0.0001 p^11^<0.0001 p^12^<0.0001	(22)

Notes: α-diversity, Species richness and evenness within individual samples; β-diversity, Diversity in species composition among different samples; Phylum → Class →Order→ Family → Genus → Species: Taxonomic ranks of the gut microbiota

rare genera*:*Clostridium XVIII, Atopobium, Holdemania, Dorea, Saccharibacteria, Delftia, Coprobacillus, Paraprevotella, Ruminococcus, Gemmiger, Akkermansia, Neisseria, Coprococcus, Fusobacterium, Methanobrevibacter, Phascolarctobacterium and Roseburia*.

In contrast, Zou et al. reported that gastric administration of ciprofloxacin induces epilepsy in rats, with gut microbiota characterized by a decrease in the phylum Firmicutes and an increase in the genus *Bacteroides* and *Akkermansia*; these changes are similar to the microbiota profiles observed in lithium-pilocarpine-induced epileptic rat models (16). However, this trend contrasts with the gut microbiota profiles of human patients with DRE. These differences may suggest that different epilepsy triggers result in distinct gut microbiota profiles. Furthermore, due to species differences, findings from animal models of epilepsy may have limitations when extrapolated to human patients. Therefore, future human studies are needed to further clarify the characteristic microbiota profiles associated with different epilepsy phenotypes and their potential mechanisms of action.

### 2.2 The Effects of the gut microbiota and its metabolites on epilepsy

Accumulating evidence indicates that dysbiosis of the gut microbiota and its metabolites can influence the onset and progression of central nervous system disorders through neural, immune, and endocrine pathways (23, 24). With respect to epilepsy, On the one hand, the increased abundance of Proteobacteria can produce lipopolysaccharide (LPS), which is significantly correlated with Spontaneous epileptic seizures after traumatic brain injury (25).They can disrupt the intestinal barrier function, increasing the central penetration of inflammatory factors and thereby heightening susceptibility to absence seizures (26–28). Meanwhile, elevated levels of circulating endotoxins (e.g., LPS) can bind to Toll-like receptor 4 (TLR4) to drive the pro-inflammatory phenotypic polarization of microglia (29), which can impairing synaptic function, thereby promoting the development of status epilepticus (SE) (30, 31).

On the other hand, the onset of epilepsy may also be associated with a reduction in beneficial gut microbiota and their metabolites. Watanangura A. et al. reported that oral treatment with phenobarbital for canine idiopathic epilepsy resulted in higher fecal SCFAs concentrations in drug-responsive group (32), suggesting the potential therapeutic effect of SCFAs in epilepsy. SCFAs are mainly produced by gut bacteria such as *Lactobacillus*, *Bacteroidetes*, Firmicutes, and *Ruminococcus* (33, 34). Animal studies have shown that rats developing spontaneous seizures after early-life brain injury exhibit a downregulation of SCFA-producing bacteria in gut with increasing intestinal inflammation and broken barrier function compared with seizure-free rats (12). SCFAs play a significant role in gut inflammation and barrier protection process. They can bind to the G protein-coupled receptor 41 (GPR41, also known as free fatty acid receptor 3, FFAR3) on the surface of CD4^+^ T lymphocytes to regulate gut immunity and exert anti-inflammatory effects (35). In case of intestinal damage, SCFAs can promote mucus production, accelerate the repair of intestinal epithelial cells (36), and enhance the expression of zonula occludens-1 (ZO-1) and claudin-1/5 to strengthen the barrier defense function (37–39). Besides intestinal barrier protection, Li et al. reported that supplementation with the SCFAs-producing bacterial genus *Lachnospira* can also alleviate blood-brain barrier damage in epileptic rats, inhibit pro-inflammatory polarization of central microglia, and reduce seizure occurrence (40).

The blood–brain barrier (BBB) dysfunction is also a key factor in epileptogenesis, which may increase the central penetration of toxic substances(such as albumin) (41). Studies have reported the associated BBB disruption in DRE patients with reduced claudin-5 expression in their brain tissues (42). Through inhibiting the histone deacetylase (HDAC)/forkhead box transcription factor O1 (FoxO1) pathway, SCFAs can upregulate claudin-5 expression (43) to enhance BBB integrity.However, the role of BBB damage in DRE still requires further study in the future.Additionally, Zhai et al. reported that butyrate can reduce hippocampal neuronal apoptosis in epilepsy, exerting direct neuroprotective effects (44).

Collectively, these studies suggest that targeted modulation of the gut microbiota and their metabolites (SCFAs, LPS) may help repair the barrier function, reduce peripheral seizure susceptibility, thus offering potential benefits for the treatment of epilepsy.

## 3 The regulatory effects of DPP-4is on gut microbiota and GLP-1 secretion: integrating the gut microbiota-SCFAs-GLP-1 axis in the antiepileptic hypothesis

Of note, DPP-4is have been demonstrated to exert consistent modulatory effects on the gut microbiota in clinical trials and animal studies of various disease models, including type 2 diabetes and fatty liver disease (45–47). They have been shown to increase the ratio of Bacteroidetes to Firmicutes in the gut (48), enhance the abundance of SCFA-producing bacteria, and promote the production of propionate and butyrate. Concurrently, they promote intestinal epithelial cell proliferation and upregulate the expression of occludin and ZO-1, thereby restoring the intestinal barrier. Furthermore, studies have reported that treating mice fed a high-fat diet with DPP-4is significantly reduced the abundance of the Proteobacteria in their intestines and significantly lowered plasma LPS concentrations (45), suggesting that DPP-4is may have an inhibitory effect on endotoxemia caused by gut dysbiosis, which maybe beneficial for lowering the seizure susceptibility. However, Smits et al. reported in a study using DPP-4is as adjunctive therapy for diabetic patients, that no significant changes in fecal microbiota were observed after 12 weeks of treatment (49), which differs from the results of gut microbiota alterations observed in multiple animal studies (48, 50). These discrepancies may be attributed to differences in observation period, small sample size(DPP-4is subgroup:18 cases), the baseline microbiota composition, the host metabolic status as well as the absence of dietary control during the experiment, which may introduce certain confounding factors to the result. In addition, interspecies differences between animal models and humans may further contribute to the inconsistency. Therefore, the modulatory effect of DPP-4is on the gut microbiota should be interpreted cautiously, and more standardized, well-controlled clinical studies are further required in the future.

**Figure 1:**
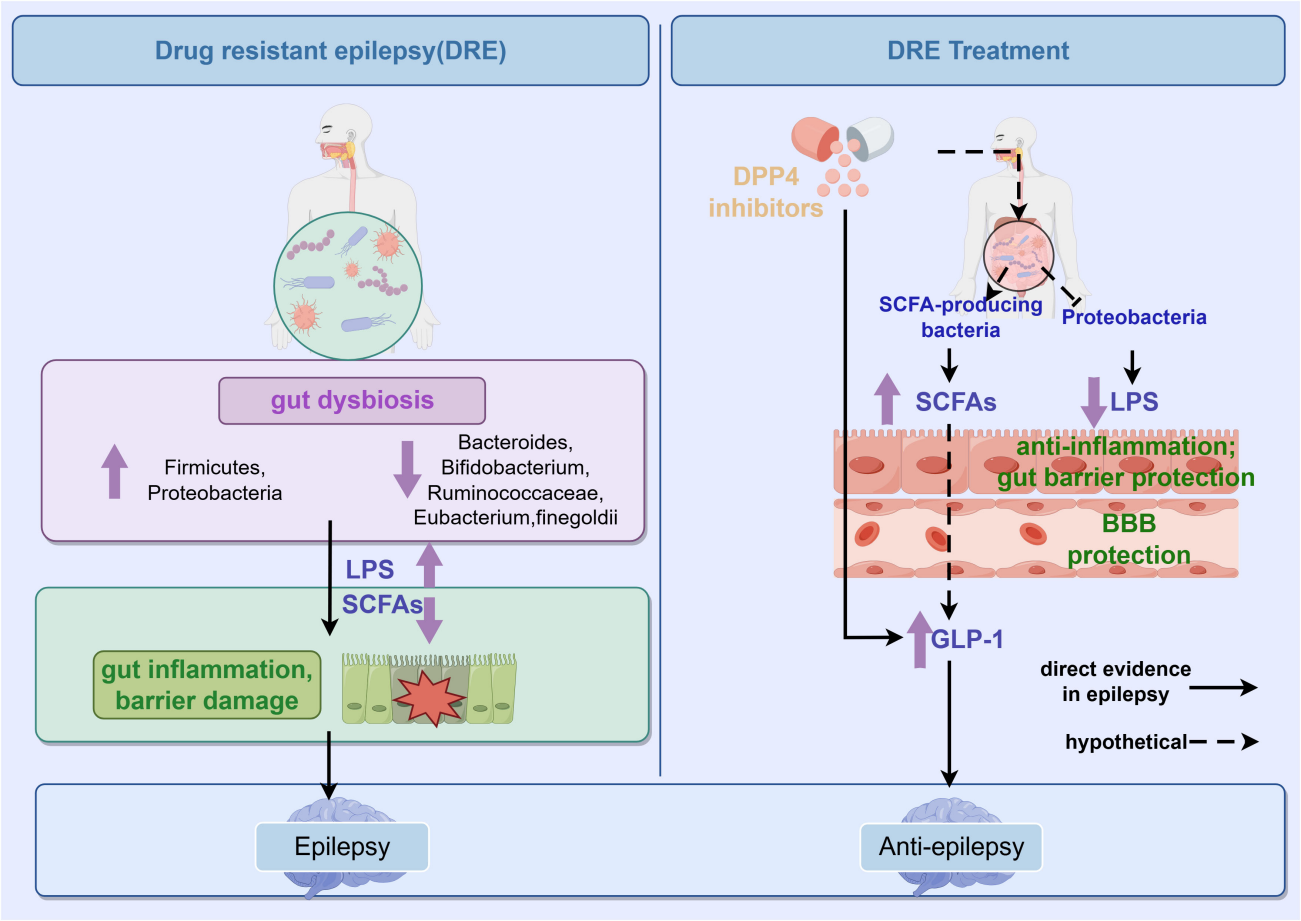
DPP-4is exert peripheral neuroprotective effects against epilepsy by modulating the gut microbiota and its metabolites, inhibiting LPS production, increasing SCFA production, and synergistically promoting GLP-1 secretion.

In addition to the modulatory effects of DPP-4is on the gut microbiota, they can also increase plasma concentration of their core substrate GLP-1. GLP-1 is a peptide hormone secreted by intestinal L cells. Carbohydrates, fats and proteins in the diet can promote its secretion. Beyond its role in regulating glucose metabolism (51), recent studies have shown that its receptor is widely distributed on the surface of central neurons and glial cells, exerting certain therapeutic effects on epilepsy (52–54), and also reducing the hepatotoxicity of ASMs (55). This suggests that regulating peripheral GLP-1 secretion is a viable strategy for the treatment and long-term prognosis of epilepsy. Under physiological conditions, DPP-4 rapidly inactivates GLP-1 by hydrolyzing its N-terminal dipeptide. The emergence of DPP-4is, by inhibiting DPP-4 activity on the surface of intestinal epithelial cells and free DPP-4 in the blood (3), significantly increase GLP-1 concentrations in both plasma and cerebrospinal fluid (56, 57), thereby extending its half-life by up to sevenfold (58), and amplifying its physiological effects potentially. Concurrently, the modulatory effects of DPP-4is on the gut microbiota increase the production of SCFAs, which act on free fatty acid receptors (FFAR2/3, also termed GPR43 and GPR41) on intestinal L cells, further promoting GLP-1 secretion (13). Studies have shown that reduced SCFAs production can inhibit the intestinal GPR43/GLP-1/GLP-1 receptor (GLP-1R) axis and further affect the function of central dopaminergic neurons (59). This suggests that SCFAs may be potential important mediators in regulating intestinal GLP-1 secretion and influencing its central effects. However, the role of the SCFAs-GPRs-GLP-1 pathway may vary depending on experimental conditions, and its exact role of controlling GLP-1 secretion in epilepsy remains to be further confirmed. In addition, the improvement of intestinal inflammation and barrier function under the influence of DPP-4is and gut-derived SCFAs (60–62) may indirectly protect the number and functional homeostasis of intestinal L cells, thereby maintaining normal GLP-1 secretion (63) (**Figure 1**). Subsequently, GLP-1 crosses the BBB via receptor-mediated transport or indirectly affects the central nervous system through the vagus nerve and neuroendocrine pathways (64). In the central nervous system, GLP-1 participates in the regulation of neural homeostasis, providing a potential basis for its anti-epileptic effects.

## 4 Central antiepileptic effects of DPP-4is/GLP-1

**Figure 2:**
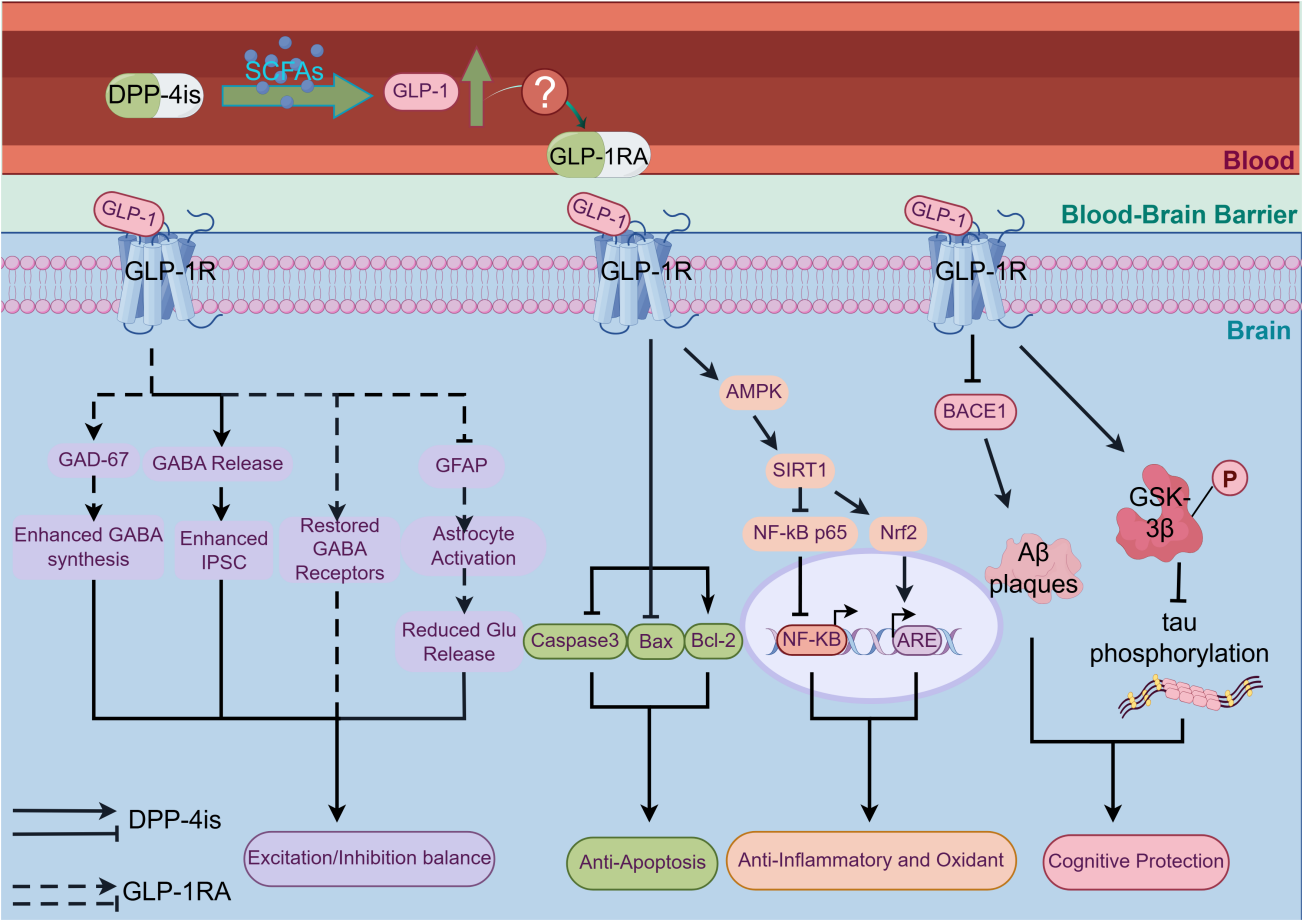
A schematic diagram of DPP-4is exert central anti-epilepsy effects via the endogenous GLP-1. This figure illustrates the diverse functions of DPP-4is/GLP-1 pathway in E/I balance, anti-apoptosis, anti-inflammatory and oxidant as well as cognitive protection in epilepsy. DPP-4is/GLP-1 suppress abnormal excitement by enhancing inhibitory synaptic transmission,modulate Glu/GABA ratio as well as the receptors. DPP-4is/GLP-1 prevent neuronal apoptosis by regulating the apoptotic pathway (Bcl-2/Bax/Caspase-3). DPP-4is exert anti-inflammatory and oxidant effects via GLP-1/AMPK/SIRT1/NF-κB/Nrf2 pathway. DPP-4is/GLP-1 reduce cognitive impairment by inhibiting BACE1 activity, suppressing Aβ deposition subsequent and inhibiting tau protein phosphorylation.

Current mechanistic studies on GLP-1 in epilepsy are predominantly based on GLP-1 receptor agonists (GLP-1RAs), while the potential antiepileptic effects of gut-derived or endogenously increased GLP-1 remain insufficiently characterized. Accordingly, this section summarizes GLP-1RA and DPP-4is related mechanisms, with a focus on DPP-4is/GLP-1-mediated central effects (**Figure 2**), to support the biological plausibility of the proposed “DPP-4is-gut microbiota-SCFAs-GLP-1 axis.”

### 4.1 Enhancement of inhibitory synaptic transmission

*γ*-aminobutyric acid (GABA)-mediated inhibitory synaptic transmission plays a critical role in regulating the excitability of neural circuits and controlling epileptic seizures. Studies have shown that the GLP-1R is widely expressed in epilepsy-related brain regions, including the cortex, hippocampus, and amygdala, and is localized at glutamatergic and GABAergic synapses (65, 66). Functional studies indicate that activation of GLP-1 signaling enhances inhibitory synaptic transmission, for example, by increasing GABAergic neuron-mediated inhibitory currents and presynaptic GABA release, as well as by enhancing GABA_A_ receptors on the plasma membrane of CA3 pyramidal neurons to increase tonic inhibitory currents (67), the latter of which hyperpolarizes the membrane potential and raises the seizure threshold. Notably, Zhang et al. reported in a febrile seizure model that DPP-4i (sitagliptin) can enhance inhibitory postsynaptic currents both *in vivo* and *in vitro (*6), and this effect can be blocked by a GLP-1R antagonist Exendin(9-39), indicating that DPP-4is primarily mediate central inhibition through endogenous GLP-1 signaling.

Furthermore, multiple studies have reported that DPP-4is (sitagliptin, alogliptin, etc.) can restore the glutamate (Glu)/GABA balance by upregulating reduced hippocampal GLP-1 levels in epileptic rats (8, 68), thereby regulating neural network excitability and exerting neuroprotective effects against epilepsy. Mechanistically, GLP-1 signaling may regulate synaptic function at multiple levels, including enhancing presynaptic GABA release, inhibiting astrocyte-mediated glutamate release, and modulating postsynaptic receptor composition, thus restoring the excitation/inhibition balance. For example, studies have reported that liraglutide, a GLP-1 analogue, upregulates the level of glutamic acid decarboxylase 67 (GAD-67) in GABAergic neurons of epileptic mice (69). GAD-67 converts glutamate to GABA, thereby increasing brain GABA concentrations (70). In addition, GLP-1RAs can inhibit overactive astrocytes in epilepsy models and downregulate the expression of glial fibrillary acidic protein (GFAP) (54), thereby reducing astrocyte-mediated glutamate exocytosis (71, 72). At the postsynaptic level, Wen et al. reported that liraglutide can restore the impaired levels of GLP-1R and GABA_A_R in the cortex of patients and rats with epilepsy, and downregulate the levels of α−amino−3−hydroxy−5−methyl−4−isoxazolepropionic acid (AMPA)- and N−methyl−D−aspartate (NMDA)-type glutamate receptors (73), thereby exerting antiseizure effects. However, the aforementioned molecular mechanisms regarding neurotransmitter metabolism and postsynaptic receptor expression are mostly derived from GLP-1RA studies, and their applicability in the context of endogenous GLP-1 modulation by DPP-4is requires further validation.

### 4.2 Anti-inflammatory and antioxidant effects

Kumar et al. performed single-cell sequencing of epileptic foci in patients with DRE and identified the presence of a pro-inflammatory microenvironment, characterized by the activation of pro-inflammatory microglia and immune cells (74). Studies have shown that abnormal excitation in neural networks during epilepsy activates the transcription of inflammatory genes in neurons and surrounding glial cells, triggering a neuroinflammatory cascade (75); Furthermore, oxidative stress is also a key contributor to the development of DRE (76), in which the free radicals can disrupt the membrane stability, causing the persistence and spread of seizure activity (77). Therefore, inhibiting central inflammation and abnormal oxidative stress is of great importance for the treatment of DRE.

Numerous animal studies have shown that GLP-1 analogues can suppress central inflammation and oxidative stress while inhibiting seizures (69, 78). GLP-1R is mainly distributed on the surface of microglia and neurons (4, 79). Zhang et al. reported downregulated GLP-1R expression in the hippocampal tissue of patients with temporal lobe epilepsy and in epileptic mouse models, accompanied by activation of pro-inflammatory microglia and the massive release of inflammatory cytokines: interleukin-1α (IL-1α) and tumor necrosis factor-α (TNF-α) (54), which further activate A1-type astrocyte, creating a pro-inflammatory central microenvironment. Administration of GLP-1R agonists Exendin-4 can reverse this process. Furthermore, DPP-4is play significant roles in anti-inflammatory and antioxidant aspects by increasing GLP-1 concentrations in the brain. El-Sayed et al. reported that treatment with the DPP-4is alogliptin can upregulate the GLP-1/adenosine monophosphate-activated protein kinase (AMPK)/silent information regulator 1 (SIRT1) pathway in the hippocampus during status epilepticus (SE) (8), thus inhibiting neuroinflammation and oxidative stress (80). Mechanistically, activation of GLP-1R promotes intracellular AMPK phosphorylation and activates its downstream NAD^+^-dependent deacetylase SIRT1 (81). Subsequently, SIRT1 can deacetylate the nuclear factor κB p65 subunit (NF-κB p65) (8, 82), thereby inhibiting the transcription of inflammatory genes such as NLR family pyrin domain containing 3 (NLRP3), interleukin-1β (IL-1β), interleukin-6 (IL-6) and upregulate the expression of anti-inflammatory molecules like interleukin-10 (IL-10) (54, 80). Simultaneously, SIRT1 can deacetylate nuclear factor erythroid 2-related factor 2 (Nrf2), promote its binding to the nuclear antioxidant response element, thereby enhancing the transcription of downstream antioxidant molecules (8), exerting central antioxidant effects.

### 4.3 Anti-apoptotic effects

Studies have shown that apoptosis is one of the key pathological features of epilepsy, particularly DRE (83). Levels of the apoptotic marker cysteinyl aspartate-specific protease (Caspase-3) are significantly elevated in the serum of children with DRE and are associated with increased seizure susceptibility (84, 85). Furthermore, Sokolova et al. performed active Caspase-3 staining on temporal lobe brain tissue from DRE patients and found that apoptosis primarily affected glial cells rather than neurons (83), suggesting that glial cell loss may be a key pathological feature of DRE foci and may further disrupt the functional homeostasis of neural networks. Therefore, targeting the regulation of glial cell apoptosis may represent an important approach for improving DRE.

Studies have shown that the DPP-4is/GLP-1 pathway can reduce apoptosis in mice with SE (8), with mechanisms involving the regulation of microglial and astrocytic states. On the one hand, DPP-4is, by upregulating endogenous GLP-1 and acting on GLP-1R on the surface of microglia, can activate the downstream AMPK/SIRT1/NF-κB pathway to inhibit the release of pro-inflammatory cytokines (IL-6, TNF-α) by microglia (8), thereby further suppressing the activation and apoptosis of A1-type astrocyte mediated by inflammatory factors (86). Concurrently, reversing the activation of microglia and astrocytes can reduce their secretion of neurotoxic proteins and pro-inflammatory factors, thus alleviating neuronal apoptosis (87, 88). On the other hand, GLP-1RA can also directly regulate endogenous apoptotic pathways in pentylenetetrazole (PTZ)-kindled mice. By acting on cellular GLP-1R, it can upregulate the expression of the anti-apoptotic protein B-cell lymphoma 2 (Bcl-2), while inhibiting the expression of Bcl-2-associated death promoter (Bax) and Caspase-3, thereby suppressing neuronal apoptosis (53, 89). Studies have shown that the use of sitagliptin can reduce apoptosis and decreases the expression of Bax and Caspase-3 in the brain of epileptic mice (90). However, whether this effect of DPP-4is depends on the activation of endogenous GLP-1 remains to be further clarified.

### 4.4 Improving cognitive deficits in epilepsy

Epileptic seizures are not only manifested as behavioral and functional disturbances caused by abnormal electrical discharges; research indicates that damage to neurons in the hippocampal CA1 and CA3 regions resulting from epilepsy can also lead to memory impairment in patients (53). However, existing surgical and pharmacological treatments for epilepsy do not significantly improve cognitive function and may further exacerbate memory decline (91). Therefore, identifying therapies that simultaneously achieve seizure control and cognitive improvement is essential for the treatment of DRE.

Previous study have shown that long-term use of DPP-4is can improve cognitive deficits in Alzheimer’s disease (AD) via GLP-1-dependent pathway (92). In epilepsy models, studies show that GLP-1RA demonstrate certain cognitive benefits through behavioral testing (53, 93), while the exact mechanism remains unclear. Safar et al. reported that sitagliptin significantly improved cognitive function in PTZ-induced epileptic rats. By upregulating GLP-1 in the brain, it inhibits the activity of beta-amyloid precursor protein cleaving enzyme 1 (BACE1), thus suppressing amyloid-β (Aβ) deposition and its associated neuronal damage (68). In addition, the treatment of DPP-4is can also promote the phosphorylation of glycogen synthase kinase-3β (GSK-3β), thus inhibiting tau protein hyperphosphorylation, which can stabilize microtubule transport. Overall, these findings suggest that DPP-4is/GLP-1 has potential and long-term value for improving epilepsy comorbidities (such as cognitive impairment) and the detailed mechanism warrants further investigation.

## 5 Clinical and translational research on DPP-4is for the treatment of epilepsy

To date, there have been no prospective clinical studies specifically evaluating the efficacy and safety of DPP-4is in patients with DRE, with most evidence derived from studies in animal models and some from diabetes-related epilepsy. In animal studies, Nader et al. found that sitagliptin exerts antiepileptic effects in a PTZ-induced epilepsy model by regulating neurotransmitter imbalances and inhibiting inflammatory responses and oxidative stress. Moreover, its combination with the antiepileptic drug pregabalin further enhances neuroprotective effects (90), suggesting that DPP-4is may have potential value as adjunctive therapy for epilepsy. However, these findings remain limited to animal models, and their clinical translational ability is unclear. A meta-analysis on epilepsy in patients with diabetes showed that the use of certain novel antidiabetic drugs, including DPP-4is, GLP-1RAs, and sodium-glucose cotransporter 2 inhibitors (SGLT2is), was associated with an approximately 24% reduction in the risk of seizures and epilepsy (94). However, further analysis indicated that this risk reduction was primarily driven by seizure events rather than epilepsy itself. This suggests that these drugs may influence neuronal excitability or triggering factors rather than directly acting on epileptogenic mechanisms. In a real-world study, a retrospective cohort analysis showed that among patients with type 2 diabetes, the risk of late-onset seizures was slightly lower in GLP-1RA users than in DPP-4is users (8-year cumulative incidence rates of 2.35% and 2.41%, respectively). Although this difference suggests that the GLP-1 pathway may play an important role in central neuroprotection, these results should be interpreted with caution given the study design and the potential confounding factors (95). In summary, the efficacy of DPP-4is in patients with DRE still requires validation through prospective studies. Clinically, DPP-4is may be most relevant for patients with DRE who have metabolic disorders, suggesting dual metabolic and neuroprotective benefits.

Beyond efficacy, potential drug interactions should be carefully considered when using DPP-4is for epilepsy. Currently, safety data on DPP-4is in patients with epilepsy remains limited. Although DPP-4is are generally considered to have a low risk of cytochrome P450-mediated interactions (96), their effects on drug transporters, including P-glycoprotein (P-gp), remain unclear. Given that P-gp is highly expressed at the BBB and plays a critical role in limiting the intracerebral accumulation of many ASMs, alterations in its activity have been strongly associated with drug resistance in epilepsy (97). Therefore, any potential modulation of P-gp function by DPP-4is could theoretically influence the brain penetration, efficacy, and toxicity profile of co-administered ASMs. Moreover, emerging evidence suggests that inflammation and gut-derived signals may regulate central P-gp expression and function (98), raising the possibility that DPP-4is, through their gut microbiota modulation and anti-inflammatory effects, might indirectly modulate BBB transporter activity. However, direct experimental or clinical evidence supporting this interaction is currently lacking. Future studies integrating pharmacokinetic analyses, BBB transport assays, and *in vivo* epilepsy models are needed to clarify whether DPP-4is affect ASMs disposition and contribute to overcoming drug resistance in epilepsy.

## 6 Discussion

Before exploring the further implications of DPP-4is in DRE, several main limitations of the current studies and this hypothesis must be acknowledged:(1)Lack of direct evidence in epilepsy: the majority of the existing evidence supporting the hypothetical “DPP-4is-gut microbiota-SCFAs-GLP-1” axis comes from cross-sectional studies (e.g., metabolic disorders, colitis, and neurodegenerative diseases), and direct causal evidence in epilepsy models is limited(**Table 2**). (2)Methodological selection bias:as this is a hypothesis-generating review, we did not conduct a systematic literature search, which may limit the completeness of the evidence synthesis. (3)Pharmacokinetic and safety interactions with anti-seizure medications (ASMs): the potential effects of DPP-4is on P-glycoprotein(P-gp) activity and BBB permeability of ASMs remain insufficiently characterized, with a lack of both mechanistic and clinical pharmacokinetic evidence. (4)Pharmacological differences between GLP-1RAs and DPP-4is:although both are incretin-based therapies. GLP-1RAs directly activate GLP-1 receptors and generally exert more potent and sustained central and peripheral effects, whereas DPP-4is indirectly enhance endogenous GLP-1 levels by inhibiting its degradation.Therefore, caution is required when extrapolating GLP-1RA-derived neuroprotective mechanisms to DPP-4is, and further studies are needed to clarify whether endogenous GLP-1 elevation can achieve comparable central effects.

**Table 2:** Casual link evidence table.

Casual link	Direct evidence in epilepsy	Model	Strength	Reference
DPP-4is→Gut microbiota	No	A fatty liver model in obese mice/ Mice fed a high-fat diet/ Patients with prediabetes/ Patients with type 2 diabetes	weak	(45–48)
Gut microbiota→SCFAs↑	Yes	cobalt wire-induced rat epilepsy model/ pentylenetetrazole (PTZ)-kindling mice model	strong	(33, 40)
SCFAs→GLP-1↑	No	mice fed fiber-free diet/ atrazine-induced PD mice model	weak	(13, 59)
DPP-4is → GLP-1→Inhibitory synaptic transmission↑	Yes	a rat model of febrile Seizures/ *in vitro* cultured rat neurons	strong	(6)
DPP-4is/GLP-1RA→Neuroprotective effects	Yes	a rat model of lithium-pilocarpine- induced Status epilepticus/ PTZ-Kindling epilepsy/PTZ-induced acute epileptogenesis in mice	moderate	(8, 68, 69, 89, 90)

Based on this, future research should focus on the following questions. (1)Whether DPP-4is simultaneously reshape gut microbiota composition, increase SCFA levels, and improve seizure outcomes in epilepsy models? (2)Is SCFA-induced GLP-1 secretion a necessary bridge for DPP-4is to exert its antiepileptic effects? (3)Well-designed clinical studies are needed to evaluate the efficacy, safety, and pharmacokinetic interactions of DPP-4is in patients with DRE.

**Figure 3:**
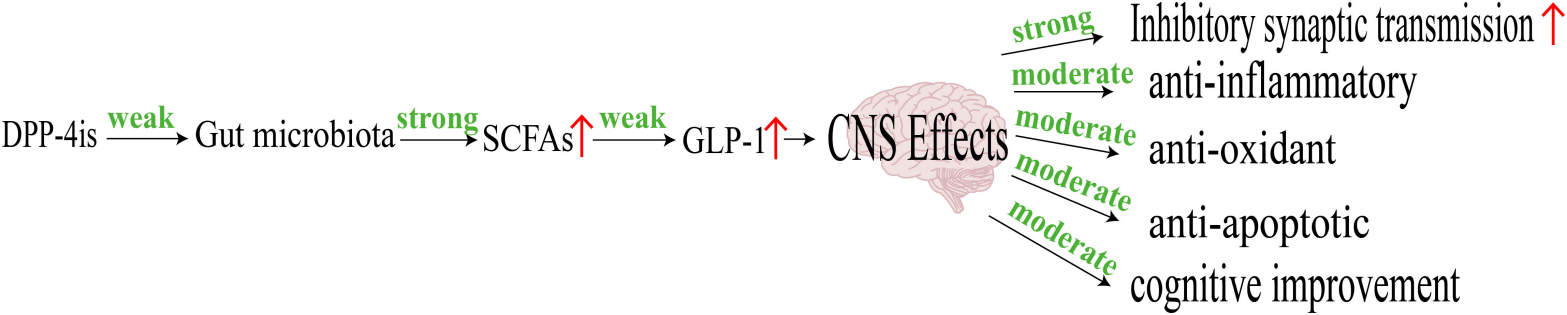
The schematic diagram of the core hypothesis(with the strength of the evidence at each step).

Despite the above limitations, the core of our hypothesis remains that in addition to the classical metabolic modulation function, DPP-4is may possess the ability to modulate gut microbiota composition and SCFA-related pathways, thus improving gut and blood-brain barrier function as well as endogenous GLP-1-mediated central neuroprotective effects(**Figure 3**). From a translational perspective, the potential value of DPP-4is is that they may provide a more specific target for modulating the SCFA-producing bacteria in epilepsy, compared with other gut microbiota interventions (such as the ketogenic diet, probiotics/prebiotics, and antibiotics), thereby providing a novel therapeutic strategy for DRE.

## Data Availability

The original contributions presented in the study are included in the article/supplementary material. Further inquiries can be directed to the corresponding author.
